# Serum HER2 levels are increased in cats with mammary carcinomas and predict tissue HER2 status

**DOI:** 10.18632/oncotarget.7551

**Published:** 2016-02-21

**Authors:** Maria Soares, Rita Ribeiro, Shabir Najmudin, Andreia Gameiro, Rita Rodrigues, Fátima Cardoso, Fernando Ferreira

**Affiliations:** ^1^ CIISA, Faculty of Veterinary Medicine, University of Lisbon, Lisbon, Portugal; ^2^ Breast Unit, Champalimaud Clinical Center, Lisbon, Portugal

**Keywords:** feline mammary carcinomas, HER2, serum HER2 levels, ELISA, dot blot assay

## Abstract

HER2 is overexpressed in about 30% of feline mammary carcinomas (FMC) and in 15-30% of breast cancers. Women with HER2-positive breast tumors are associated with shorter survival. This study aimed to optimize the detection and quantification of serum HER2 (sHER2) in cats and to evaluate its potential in diagnosing cats with mammary carcinomas (MC) overexpressing HER2. A prospective study was conducted in 60 queens showing MC and 20 healthy animals. Pre-operative serum samples were collected for sHER2 quantification using two immunoassays: ELISA and Dot blot assay. sHER2 levels were compared with tissue HER2 status assessed by immunohistochemistry. Queens with FMC showed significantly higher mean levels of sHER2 by both ELISA and Dot blot assay. A significant difference in the sHER2 levels was also found between cats with HER2-positive MC and those with low-expressing HER2 MC. A significant correlation between sHER2 levels and tumor HER2 status was also found, particularly when ELISA was used (*r* = 0.58, *p* < 0.0001). The value of 10 ng/ml was proposed as the optimal cutoff for both immunoassays by ROC analysis. Like in humans, sHER2 levels are increased in cats with MC HER2-positive, strongly suggesting that evaluation of sHER2 levels can be very useful in feline oncology. The results show that ELISA and Dot blot assay can replace the immunohistochemistry technique, due to their efficacy and lower costs for diagnostic purposes and for monitoring the response to anti-HER2 therapies in cats.

## INTRODUCTION

Animal models have been crucial in increasing our understanding of tumorigenesis, with mouse species being the most commonly used because of their small size and short gestation period [1]. However, due to several limitations in using laboratory rodents, prevailing tumors in pets appear to be good alternative models, especially because of their high incidence. Moreover, pets also share a similar environment to humans and show a more similar body size, thus facilitating the pharmacokinetic and toxicological studies and shortening the phase I trials in humans [1-5].

Feline mammary carcinomas (FMC) are very common in cats, showing an incidence that ranges from 12 to 40% of all tumors [6, 7]. Their epidemiological and histopathological features closely resemble those found in the more aggressive breast cancer types [8]. Thus, it has been proposed as a putative model for cancer studies [2, 8-13]. Recently, some reports have shown that the feline homologue of human epidermal growth factor receptor-2 proto-oncogene (HER2) is overexpressed in 33%-60% of FMC [10, 13, 14, 15] and it was associated with a shorter overall survival (OS) [14] as in human HER2 positive breast tumors, even though the HER2 gene amplification could not be detected [10, 13].

HER2 has a molecular mass of 185 kDa and is a transmembrane glycoprotein which comprises three domains: an extracellular domain (ECD), a short transmembrane region and an intracellular domain with tyrosine kinase activity [16-18]. In women, the gold standard method to identify HER2-positive breast tumors is immunohistochemistry (IHC), with fluorescence in situ hybridization (FISH) used additionally to identify HER2 gene amplification status in ambiguous cases [19, 20]. Nevertheless, several limitations, like the impossibility of conducting continuous follow-up after the invasive surgery and the high costs of the reagents, has led to the development of new non-invasive techniques to quantify HER2 in serum (sHER2). The HER2-ECD is shed from the surface of tumor cells into the bloodstream via protease activity (A Disintegrin And Metalloproteinase domain-containing proteins - ADAMs family), allowing its detection in sera by ELISA or by a chemiluminescence method [21]. In fact, many studies have found that breast cancer patients with elevated HER2-ECD levels were associated with higher relapse rates and worse prognosis [21-23]. More recently, it was reported that the Dot blot assay could also be used to measure sHER2 levels, being a less expensive method and representing a good alternative to closely follow the tumor disease progression [21, 24, 25].

To the best of our knowledge, no studies have been published on the utility of assessing sHER2 levels in small animals. Thus, the main objectives of this study were: i) to accurately quantify the sHER2 levels in cats using both ELISA and the Dot blot assay; ii) to evaluate the usefulness of measuring sHER2 levels in the diagnosis of FMC overexpressing HER2; and iii) to determine the optimal cutoff value for the ELISA and the Dot blot assay, that would differentiate cats with mammary carcinoma overexpressing HER2 from cats with HER2-negative mammary carcinomas or healthy animals.

## RESULTS

### Animal study population

A total of sixty queens displaying mammary carcinoma were used in the study. Their main clinicopathological characteristics are summarized in Table 1. The mean age at diagnosis was 11.57 years (range, 7-17 years) and all animals were treated surgically, except one, which displayed pulmonary metastasis. Fifty (83.3%) cats were subjected to unilateral mastectomy, six (10%) to bilateral mastectomy and the remaining 3 (6.7%) to regional mastectomy. After surgery, five of these queens were subjected to anthracycline-based adjuvant chemotherapy (doxorubicin, 25 mg/m^2^, intravenously, every 3 weeks for 5 cycles). Tumor samples from the untreated animal were collected after euthanasia.

**Table 1:** Clinicopathological features of 60 female cats with mammary carcinoma

Clinicopathological feature	Number of animals (%)	Clinicopathological feature	Number of animals (%)
**Breed**		**Size**	
Not determined	46 (76.7%)	<2 cm	24 (40.0%)
Siamese	9 (15.0%)	2-3 cm	24 (40.0%)
Persian	3 (5.0%)	>3 cm	12 (20.0%)
**Norwegian Forest Cat**	**2 (3.3%)**	**^a^HP classification**	
Spayed		Tubulopapillary carcinoma	36 (60.0%)
No	30 (50.0%)	Solid carcinoma	13 (21.7%)
Yes	29 (48.3%)	Cribriform carcinoma	6 (10.0%)
Unknown	1 (1.7%)	Mucinous carcinoma	5 (8.3%)
**Contraceptives**		**Malignancy grade**	
No	18 (30.0%)	I	3 (5.0%)
Yes	31 (51.7%)	II	14 (23.3%)
Unknown	11 (18.3%)	III	43 (71.7%)
**Treatment**		**Necrosis**	
None	1 (1.7%)	No	20 (33.3%)
Mastectomy	54 (90.0%)	Yes	40 (67.7%)
Mastectomy + Chemo	5 (8.3%)	**Lymphatic invasion**	
**Multiple tumors**		No	49 (81.7%)
No	22 (36.7%)	Yes	11 (18.3%)
Yes	38 (63.3%)	**Lymphocytic infiltration**	
**Lymph node status**		No	24 (40.0%)
Negative	32 (53.3%)	Yes	36 (60.0%)
Positive	23 (38.3%)	**Tumor ulceration**	
Unknown	5 (8.3%)	No	57 (95.0%)
**Stage (TNM)**		Yes	3 (5.0%)
I	13 (21.7%)	**Ki 67 index**	
II	9 (15.0%)	Low (< 14%)	24 (40.0%)
III	29 (48.3%)	High (≥ 14%)	35 (58.3%)
IV	9 (15.0%)	Unknown	1 (1.7%)
**Localization**		**PR status**	
M1	9 (15.0%)	Negative	28 (46.7%)
M2	12 (20.0%)	Positive	32 (53.3%)
M3	24 (40.0%)	**ER status**	
M4	14 (23.3%)	Negative	40 (33.3%)
Unknown	1 (1.7%)	Positive	20 (66.7%)
		***f*HER2 status**	
		Negative	38 (63.3%)
		Positive	22 (36.7%)

a HP classification, Histopathological classification

The mean size of the primary mammary carcinomas was 2.33 cm (range 0.3-7 cm) and the mean value of Ki-67 proliferation index was 19.5% (ranging between 1.2% and 46%). Twenty two (36.7%) cats showed HER2-overexpressing MC (assessed by IHC), out of which nine (15%) were classified with a 3+ and thirteen (21.7%) with a 2+ score (Table 1).

Survival data for disease free-survival (DFS) were available for 52 queens. Sixty-four percent (33/52) of the cats with mammary carcinoma showed disease recurrence at the end of the follow-up period (2 years and 2 months), with the majority showing locoregional relapses (24/33, 72.7%) and the rest distant metastases (27.3%, 9/33). The mean DFS was 13.4 ±1.82 months (95% CI: 9.8-17 months). Regarding the overall survival (OS), data were available for 58 animals, from which twenty-nine (50%) died, presenting a mean survival of 27.18 ±3.4 months (95% CI: 20.5-33.9 months).

### Cats with mammary carcinoma show elevated sHER2 levels

**Figure 1:**
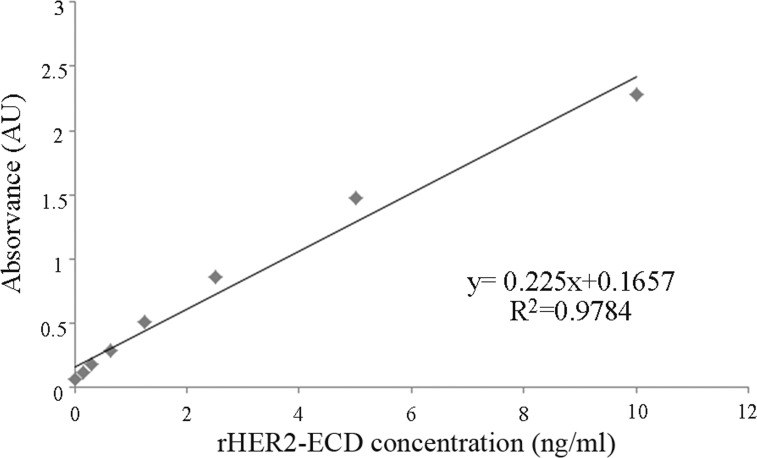
Standard curve for sHER2 measurements using a commercial ELISA kit The intra and inter-assay coefficients of variation were < 10%.

Serum HER2 levels were measured by both ELISA and Dot blot assay. The circulating HER2 levels were calculated from the standard curve of known rHER2-ECD concentrations (Figure 1). The intra-assay and inter-assay coefficients of variation were 6.2% and 4.6%, respectively.

Box plots were used to illustrate the full data range of each group and to identify outliers. Cats having sHER2 levels that fall more than three standard deviations away from the mean were considered outliers and removed from further analysis. ELISA results gave two outliers in the healthy group and seven in the cancer group that were removed. There were no outliers in the Dot blot results.

**Figure 2:**
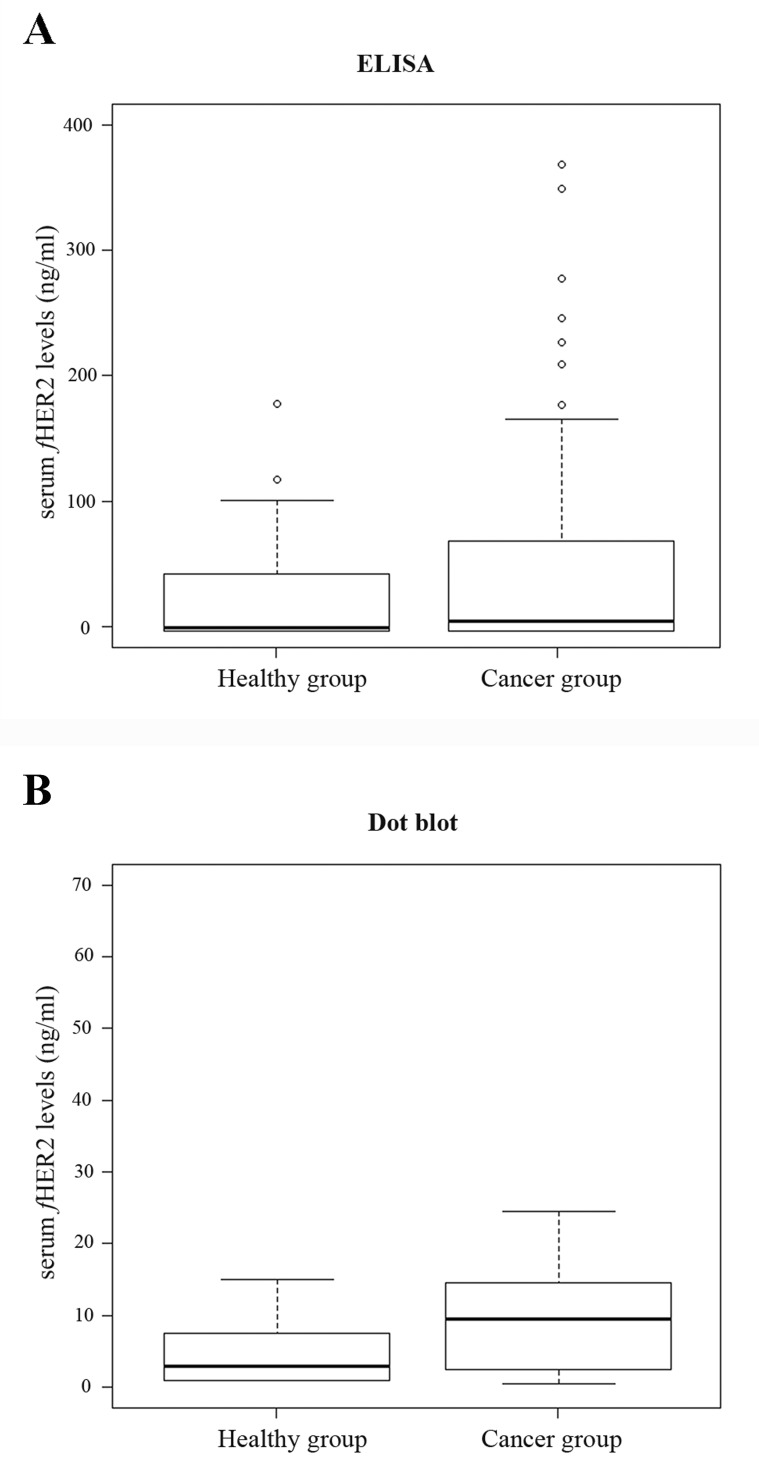
Box plot diagrams representing the sHER2 levels in control cats (healthy group) and in cats with mammary carcinoma (cancer group) determined by ELISA (A) and Dot blot assay (B) Cats with mammary carcinomas had significantly higher mean of sHER2 levels than healthy cats both by ELISA (*p* = 0.04) and Dot blot assay (*p* = 0.01). Outliers are indicated by open circles.

ELISA results revealed that cats from the cancer group showed significantly higher sHER2 levels (mean = 22.3 ng/ml; range of values: 0-147.05 ng/ml) than healthy animals (mean = 18.3 ng/ml; range of values: 0-104.2 ng/ml), with a significant *p*-value of 0.04 (Figure 2A). A more significant difference between the sHER2 levels of the two animal groups was found when Dot blot assay was performed (*p* = 0.01), with the cancer group showing a mean value of sHER2 of 14 ng/ml (range of values: 0-75 ng/ml) and the healthy group a mean value of 4.5 ng/ml (range of values 0-15 ng/ml), as depicted in Figure 2B.

### Serum HER2 levels predict the tumor HER2 status

**Figure 3:**
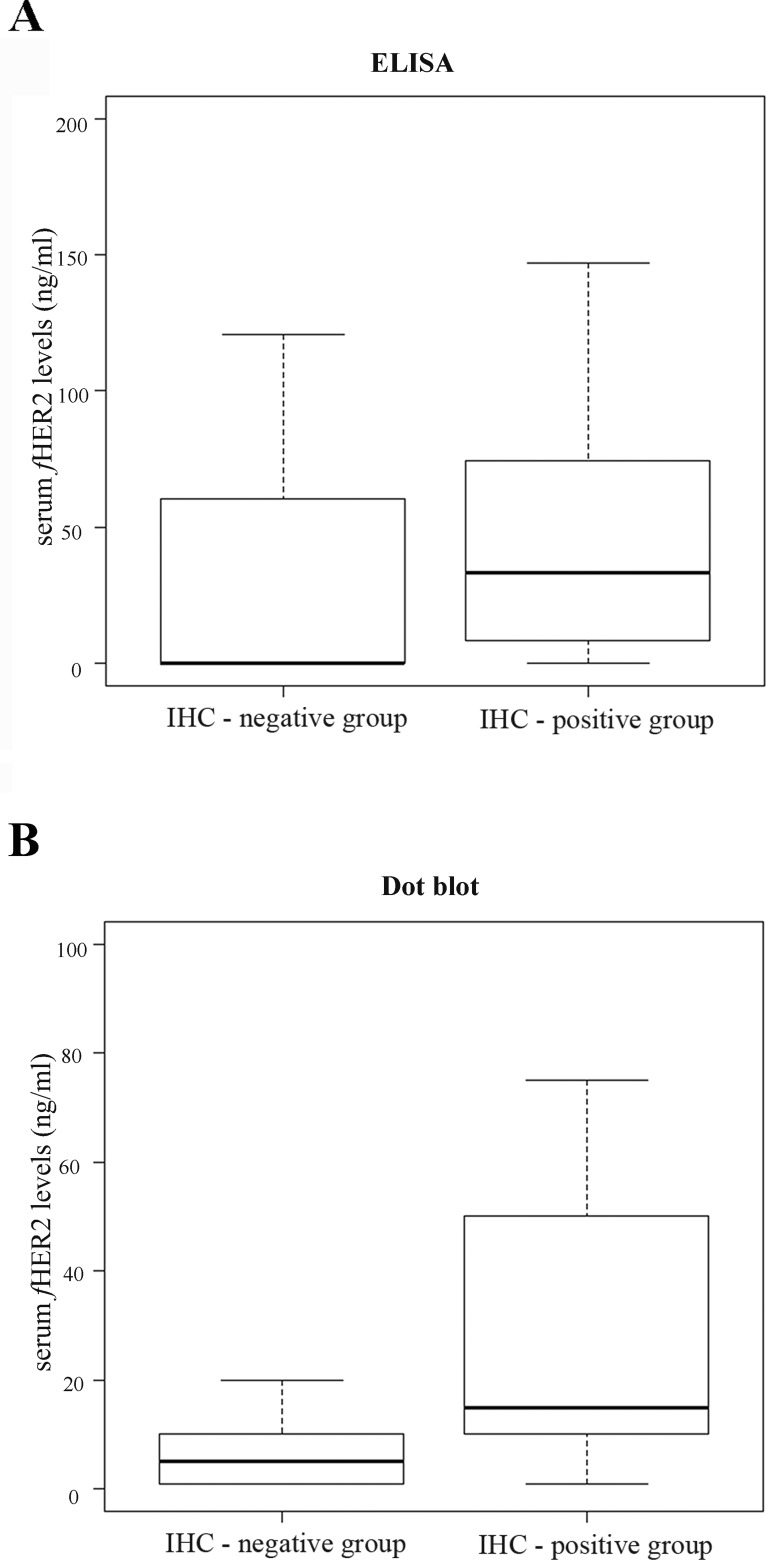
Box plot diagrams showing that tumor HER2 status correlates with sHER2 levels as assessed by both ELISA (A) and Dot blot assay (B) **A.** A significant difference was found (*p* = 0.001) between the sHER2 levels of cats with MC overexpressing HER2 (IHC-positive group: mean 42.6 ng/ml [range of values 0-147.05 ng/ml]) and the sHER2 levels of cats with HER2-negative MC (IHC-negative group: mean 8.8 ng/ml [range of values 0-73.89 ng/ml]), using ELISA (*p* = 0.001). **B.** The Dot blot assay also showed a significant difference between the sHER2 levels measured in these two studied groups, with a *p-*value of 0.03 (IHC-positive group: mean 24.4 ng/ml [range of values 0-75 ng/ml]; IHC-negative group: mean 8 ng/ml [range of values 0-20 ng/ml]).

A significant difference was found between sHER2 levels of cats with HER2-negative mammary carcinoma (IHC-negative group) and sHER2 levels of cats diagnosed with mammary carcinoma overexpressing HER2 (IHC-positive group), by both ELISA (*p* = 0.001, Figure 3A) and Dot blot assay (*p* = 0.03, Figure 3B). Additionally, a strong correlation was found between sHER2 levels quantified by ELISA and tumor HER2 status (r = 0.58, *p* < 0.0001), coupled with a moderate association between sHER2 levels measured by Dot blot assay and tumor HER2 status (r = 0.26, *p* < 0.1). Moreover, the Kappa coefficient showed a moderate agreement between ELISA and IHC results (k = 0.48, *p* = 0.002), and a good agreement between Dot blot assay and IHC results (k = 0.264, *p* = 0.047). However, results obtained by ELISA and Dot blot assay only showed a fair agreement (k = 0.27, *p* = 0.048).

### Serum HER2 molecules contain a portion of the intracellular receptor domain

**Figure 4:**
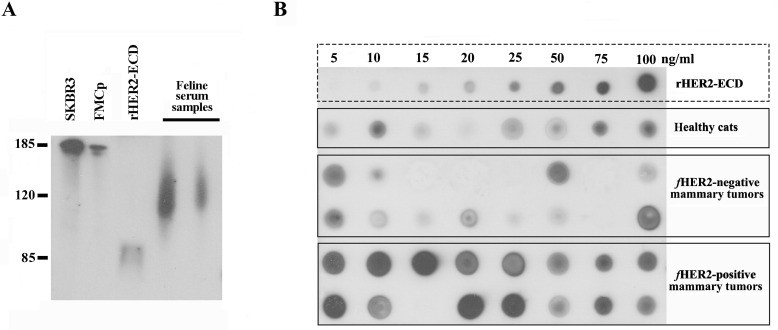
Soluble truncated HER2 forms carry a portion of the ICD and are quantifiable by Dot blot assay **A.** The anti-HER2 SP3 antibody specifically recognizes a protein band of about 185 kDa in human and feline whole cell extracts (SKBR3 and FMCp, lanes 1 and 2), the purified rHER2-ECD (lane 3, ∼75 kDa), and a protein with a molecular weight that ranges from 100 to 140 kDa in cat serum samples (lanes 4 and 5). **B.** A representative image of an immunoblot performed with serum samples collected from healthy cats (line 2) and cats with mammary carcinoma (lines 3-6). sHER2 levels were semi-quantified by comparing the intensity of each serum dot with the intensities of the dots obtained from rHER2-ECD dilutions (line 1).

In order to validate the measurement of sHER2 levels by the Dot blot assay, the antigenic specificity of the anti-HER2 monoclonal antibody (clone SP3) was evaluated through western blot analysis. As expected, the SP3 antibody recognizes a protein band of ∼185 kDa corresponding to predicted molecular weight of full-length HER2 in whole cell extracts of both human breast cancer cells (SKBR3, Figure 4A, line 1) and feline mammary tumor cells (FMCp, Figure 4A, lane 2), and cross-reacts with the recombinant human HER2-ECD (Figure 4A, lane 3, 84-90 kDa). Further western blot analysis revealed that the anti-HER2 monoclonal antibody detected several protein bands with masses ranging from 120 to 140 kDa in the serum samples from cats diagnosed with MC overexpressing HER2 (Figure 4A, lanes 4 and 5), suggesting that proteolytic cleavage of HER2 occurs at the intracellular domain (ICD), leading to the production of truncated HER2 soluble forms which are quantifiable by the Dot blot assay (Figure 4B). As mentioned above, cats diagnosed with HER2-overexpressing MC (scored as 2+ and 3+), showed significantly (*p* = 0.03) higher sHER2 levels (Figure 4B, lines 5 and 6) than cats with HER2-negative mammary carcinomas (Figure 4B, lines 3 and 4) or healthy cats (Figure 4B, line 2).

### sHER2 levels ≥10 ng/ml give the optimal cutoff value to diagnose HER2-overexpressing FMC

**Figure 5:**
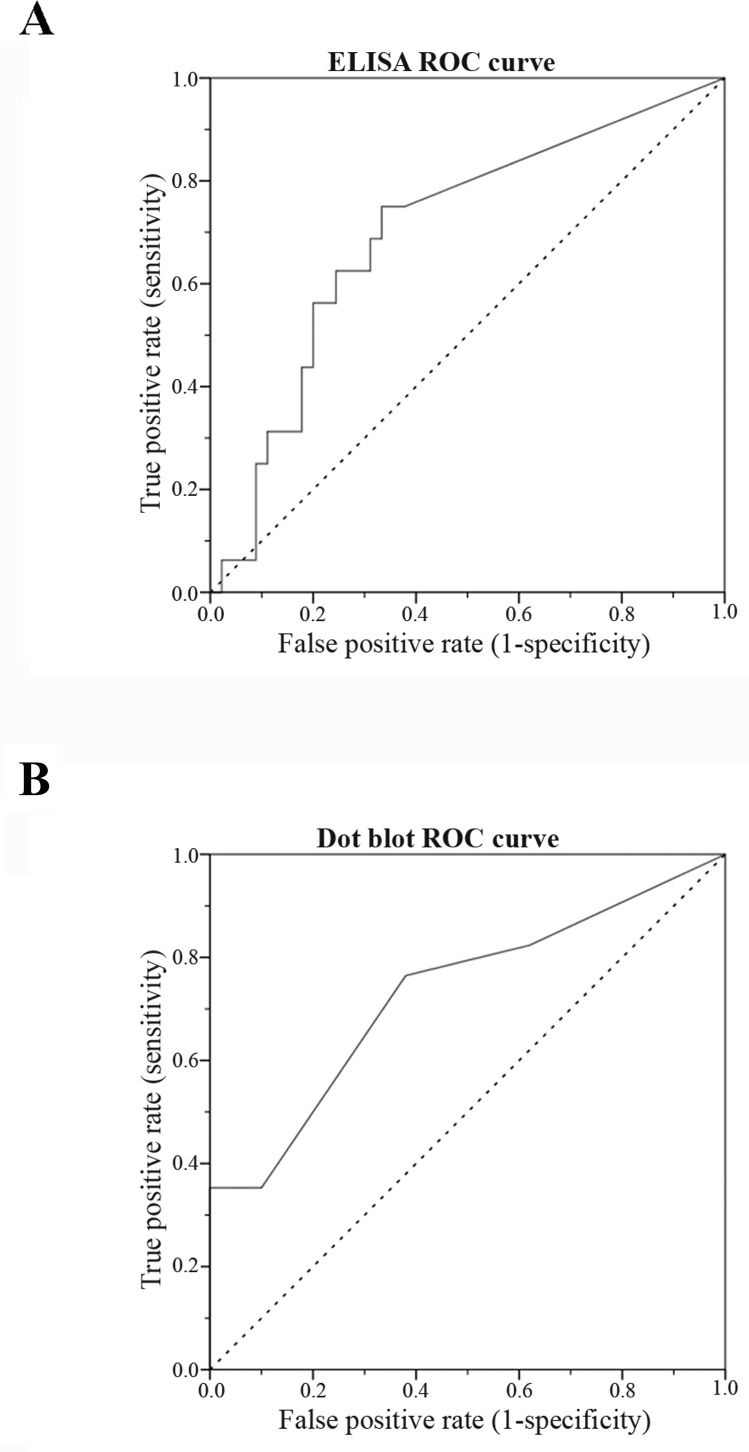
Receiver-operating characteristic (ROC) curve for sHER2 levels for ELISA (A) and Dot blot assay (B) The optimal sHER2 cutoff value (10 ng/ml) was chosen to maximize the sum of the sensitivity and specificity on the Youden index (sensivity+specifity-1). The AUC was 0.70 (95% CI: 0.55-0.85) for ELISA **A.** and 0.73 (95% CI: 0.58-0.88) for Dot blot assay **B.**, demonstrating that the sHER2 levels are a good biomarker to differentiate cats with MC overexpressing HER2 from cats with MC non-expressing HER2 or healthy cats. When the cutoff value of 10 ng/ml was set, the sensibility was 69% and the specificity was 67% for ELISA, whilst for Dot blot assay the sensibility and the specificity were 53% and 78%, respectively.

To determine the best cutoff value, the sensitivity and the specificity of both ELISA and Dot blot assay were determined using different cutoff points to classify cats according to their sHER2 levels. ROC curve analysis revealed that the best cutoff was ≥10 ng/ml to discriminate cats with mammary carcinomas overexpressing HER2 (Table 2). By using this threshold value, the sensitivity and the specificity of ELISA were 69% and 67%, respectively (Figure 5A, [AUC = 0.70, 95% CI = 0.55-0.85]), while the Dot blot assay revealed a sensitivity of 53% and a specificity of 78% (Figure 5B, [AUC, 0.73; 95% CI = 0.58-0.88]).

**Table 2:** Serum HER2 levels in cats with mammary carcinoma

Immunoassay	Cats (n, %)	Mean and range values (ng/ml)
**ELISA (*n* = 40)**		
Low levels (< 10 ng/ml)	24 (60.0%)	0.35 (0.00-8.38)
High levels (≥ 10 ng/ml)	16 (40.0%)	55.17 (13.75-147.05)
**Dot blot assay (*n* = 47)**		
Low levels (< 10 ng/ml)	20 (42.6%)	2 (0-5)
High levels (≥ 10 ng/ml)	27 (57.4%)	22.78 (10-75)

Six out of twenty healthy cats (30%) had elevated sHER2 levels (≥10 ng/ml) when ELISA was used and 5/20 (25%) with the Dot blot assay. This is in accordance with studies in humans, where false-positive rates can reach up to 20% [25].

### Elevated sHER2 levels are associated with a less aggressive tumor phenotype

ELISA results revealed that cats with sHER2 levels ≥10 ng/ml were significantly associated with the early stage of the disease (stage I, *p* = 0.006; Odds Ratio [OR] = 17.9; 95% CI: 1.633-984.42), absence of tumor necrosis (*p* = 0.0024; OR = 9.28; 95% CI: 1.79-60.61) and lower Ki-67 values (< 14%, *p* = 0.007; OR = 7.73; 95% CI: 1.57-47.54). In parallel, the Dot blot assay results also correlate with high sHER2 levels (≥10 ng/ml) with cats that have mammary carcinomas with low Ki-67 index (*p* = 0.016; OR = 5.24; 95% CI: 1.23-27.79). Supporting the above results, significant correlations were found between the HER2-negative tissue status and the presence of tumor necrosis (*p* = 0.0012; OR = 7.16; 95% CI: 1.91-30.5), moderate malignancy grade (*p* = 0.049; OR = 3.4; 95% CI: 0.8-14.6), low Ki-67 values (*p* = 0.0022; OR = 6.42; 95% CI: 1.81-25.5) and ER positivity (*p* = 0.049; OR = 3.15; 95% CI: 0.91-11.45).

## DISCUSSION

In this study, 36.7% (22/60) of the selected population had HER2-overexpressing mammary carcinomas, corroborating previously published data [8, 10, 13, 14].

To the best of our knowledge, this is the first work to detect and quantify the serum HER2 levels in cats, using two immunoassays: the ELISA which has already been approved to measure sHER2 levels in women with metastatic breast cancer, and the Dot blot assay, a less expensive technique [24, 26]. Our results show that sHER2 levels in cats with HER2 overexpressing mammary carcinomas are significantly higher than in cats with HER2-negative mammary carcinomas (*p* = 0.001) or healthy animals (*p* = 0.04). As also reported for breast cancer patients, a false-positive rate of 25-30% was found in healthy cats, when either ELISA or Dot blot assay was performed [21, 25, 27].

Using ROC analysis the best cutoff value to identify cats with mammary carcinomas overexpressing *f*HER2 was 10 ng/ml in both techniques. Interestingly, this threshold is also used in woman with breast cancer [25, 28]. Comparing the two assays, ELISA was more sensitive in detecting cats with elevated sHER2 levels than Dot blot assay (69% versus 53%), but less specific (67% *versus* 78%).

Moreover, a moderate correlation was found between the sHER2 levels and the tissue HER2 status when ELISA was employed (r = 0.58, *p* < 0.0001), revealing that sHER2 levels predict the HER2 status of the primary mammary tumor. However, the correlation with the Dot blot assay was low (r = 0.3). ELISA studies in humans showed contradictory results: some did not find any correlation between tissue and serum HER2 levels [29], whereas others reported a poor or a moderate/strong correlation [21, 23, 25, 30]. Regarding the Dot blot assay technique, there is only one study that correlates the tissue and serum HER2 levels, giving a similar result to this study (r = 0.3) [24].

Western blot analysis demonstrated that the soluble HER2 fragments also contain a portion of the intracellular domain, since a protein band between 100 and 135 kDa was detected. This result was additionally supported by the use of two anti-HER2 antibodies that recognize the intracellular domain of HER2 (4B5 and A0485) in the Dot blot assay, where a similar signal intensity to the SP3 antibody was obtained.

Further statistical analysis revealed that cats with MC showing HER2 overexpression or high sHER2 levels were associated with features usually associated with a better outcome (low Ki-67 index, absence of tumoral necrosis, ER-positive status and lower disease stages). Previous studies in cats did not show significant associations [9, 14, 31]. Although HER2-positive tumors are associated with aggressive tumoral features [21, 23, 32], our results could be due to the fact that most of HER2-overexpressing FMC also presented a positive ER and/or PR status, being classified as luminal B and not as HER2-positive immunophenotype. Indeed, luminal B breast tumors in humans showed a less aggressive behavior than HER2-positive tumors [33].

In summary, this study revealed that cats with mammary HER2-overexpressing carcinomas showed elevated sHER2 levels, which can be detected and quantified by ELISA and Dot blot assay. Even though more studies are needed to clarify the role of HER2 in the pathogenesis of FMC, our results showed that sHER2 level measurements are useful to diagnose FMC overexpressing HER2, and probably, to predict the therapeutic response to anti-HER2 agents (antibodies or kinase inhibitors), as in humans, where a rapid decrease of the sHER2-ECD levels after treatment is indicative of a good therapeutic response [21, 34, 35].

This study has also shown that *f*HER2 protein can release its extracellular domain into the extracellular space, an event that is apparently similar to the one described in humans. ELISA appears to be the best technique, giving a higher correlation with the tissue level and although less specific than Dot blot assay, could be a supplement to the IHC with possible applications in Veterinary Medicine. High serum *f*HER2-ECD levels are present in 40% of the feline patients (using the ELISA assay) and overexpression of *f*HER2 protein was observed in 36.7% of the population (with IHC).

Surprisingly, the *f*HER2 protein in serum and in tissue was associated with less aggressive features, contradicting what is described for humans. These findings, together with the non-amplification of the *f*HER2 gene [13], reinforce the need for more studies in order to clarify the biological role of this protein.

Finally, the high prevalence of this protein in FMC could be important for the study of specific target therapies directed against HER2 protein, both in a veterinary clinical perspective, improving the therapeutic options of these animals and also in a comparative perspective, using the cat as a model for therapeutic trials.

## MATERIALS AND METHODS

### Cat study population

Sixty female cats with spontaneous mammary carcinomas that underwent surgical treatment at the Small Animal Hospital of the Veterinary Medicine Faculty, University of Lisbon, were selected in a prospective study from June 2011 to September 2013. For each animal, the following clinicopathological features were recorded: age, breed, reproductive status, progestogens administration, number, location and size of tumor lesions, performed treatment (none, surgery, surgery plus chemotherapy), histopathological classification, malignancy grade, presence of tumor necrosis, lymphatic invasion by tumor cells, lymphocytic infiltration, cutaneous ulceration, regional lymph node involvement, stage of the disease (TNM system) [36], DFS and OS.

Excised mammary glands, mammary tumors and regional lymph nodes from the animals were immediately fixed in 10% formalin neutralized with 0.1 M phosphate buffer (pH 7.2), during a period no longer than 48 hours. All samples were embedded into paraffin blocks and serial histological sections of 3 μm thickness were prepared, prior to hematoxylin and eosin staining. Carcinomas are classified according to the WHO system adapted by Misdorp et al., 1999 [37] and the degree of malignancy was assessed according to the Elston and Ellis grading system [37, 38], which classifies tumors into grade I (well differentiated), grade II (moderately differentiated), and grade III (poorly differentiated).

### Tissue HER2, ER and PR status and Ki-67 assessment

A representative area of each FMC with a diameter of 0.6 cm was selected and tissue sections of 3μm thickness were mounted on glass slides (Star Frost adhesive glass slides, Thermo Scientific, Rockford, USA), deparaffinized with xylene and hydrated in a graded ethanol series to distilled water. For HER2, ER and Ki-67 immunostaining, antigen retrieval was performed by immersing glass tissue slides in citrate buffer (0.01M NaCH_3_COO, pH 6.0) and using a pressure cooker (2 min at 2 atm), while for PR immunodetection, an immersion in water bath (60 min at 95°C) was performed [13]. Slides were cooled for 10 min at room temperature and rinsed twice for 5 min in triphosphate buffered saline (TBS). Endogenous peroxidase activity was blocked with Peroxidase Block Novocastra (Novocastra, Newcastle, UK) for 5 min. Tissue samples were then incubated at 4°C overnight, in a humidified chamber, with the following primary antibodies: mouse anti-HER2 (clone CB11, 1:200, Invitrogen, Carlsbad, CA, USA), mouse anti-ER (clone 6F11, 1:125, Thermo Scientific), rabbit anti-PR (clone 1E2, ready-to-use, Ventana, Tucson, USA) and rabbit anti-Ki-67 (polyclonal, 1:500, Thermo Scientific). The staining was performed using a modified streptavidin-peroxidase conjugate method based on the poly-HRP anti-rabbit IgG detection system (Novolink MaxPolymer Detection System, Leica Biosystems, Wetzlar, Germany), following the manufacturer's guidelines. Finally, tissue sections were counterstained with Mayer's hematoxylin (Merck, New Jersey, USA). HER2 immunoreactivity was scored according to the American Society of Clinical Oncology's recommendations [19], as summarized in Table 3. Briefly, FMC were classified as HER2-negative when scored 0 or +1 and HER2-positive if scored as +2 or +3 [13, 39]. Mammary carcinomas were also evaluated for ER/PR status using the Allred score system on a scale of 0 to 8 (Table 4), and only tumors with a score ≥ 2 were considered positive [40-42]. The Ki-67 proliferation index was determined by dividing the number of tumoral cells showing positive nuclear immunostaining per 1000 tumor cells analyzed over at least three high-amplified microscopic fields [43]. Tumors were considered highly proliferative when more than 14% of the neoplastic cells nuclei expressed Ki-67 [43, 44].

**Table 3:** HER2 immunohistochemistry scoring criteria

Score	Interpretation
0	No staining
+1	Weak, incomplete membrane staining in any proportion of tumor cells
+2	Complete membrane staining that has either no uniform or is weak in intensity, but with obvious circumferential distribution in at least 10% of cells
+3	Uniform intense membrane staining of at least 10% of invasive tumor cells

**Table 4:** IHC semi-quantitative scoring system for ER/PR assessment [41, 42]

Proportion of positive staining tumor cells		Average staining intensity	
Score	Interpretation	Score	Interpretation
0	No staining	0	None
1	<1%	1	Weak
2	1-10%	2	Average
3	10-33%	3	Strong
4	33-66%		
5	>66%		
**Allred score (0-8)** = proportion of positive staining tumor cells (0-5) + average staining intensity (0-3)			

Finally, histological samples of feline mammary carcinomas with previous known ER/PR/HER2 status were used as controls, whereas a feline tonsil tissue sample was used as a positive control for the assessment of Ki-67 index, according to the manufacturer's instructions.

All slides were independently subjected to blind scoring by two independent pathologists. Discordant interpretations were further debated and settled using a multiobserver microscope.

### Quantitative immunoassays to measure sHER2 levels

A blood sample was collected from all the sixty queens with mammary carcinoma and from twenty healthy queens presented for elective ovariohysterectomy. The serum was separated from clotted blood by centrifugation (1500 g, 10 min, 4°C), stocked in small aliquots (100 μL) and stored at −80°C.

Considering the extensive sequence homology between human HER2 receptor and its homologue in *Felis catus* (93%), sHER2 levels in cats were evaluated by using a commercial ELISA-based kit, suitable to measure sHER2 levels in humans, and also by an optimized Dot blot assay procedure. Measurements were performed in a blind manner, without knowing IHC results and discarding all feline blood samples that show hemolysis (n = 13), as recommended for humans [45].

### Enzyme-linked immunosorbent assay (ELISA)

Serum HER2 levels were measured in 67 cats (47 ill and 20 healthy) using an ELISA sandwich assay (sHER2 Platinum ELISA kit, eBioscience, San Diego, USA), following the manufacturer's recommendations. A standard curve was generated using seven solutions of recombinant human HER2-ECD (rHER2-ECD) with known concentrations (0.16, 0.31, 0.63, 1.25, 2.5, 5 and 10 ng/ml). Briefly, first row of a 96-well ELISA plate was coated with 100 μl/well of each rHER2-ECD dilution, in duplicate, on “standards wells”, whereas 10 μl of each serum sample was added to 90 μl of assay buffer in “sample wells”, also in duplicates. After two consecutive washes (2×300μl with Wash Buffer), 50 μl of an HRP-conjugated mouse anti-IgG was added to each well and incubated at 37°C, for 2 hours, on a microplate shaker at 100 rpm. After a second washing step (3×300 μl), 100 μl of the 3,3′,5,5′-tetramethyl-benzidine (TMB) substrate solution was added to each well and the final mixture was incubated at RT, for 10 min, in the dark. The reaction was interrupted by adding 100 μl of stop solution per well and absorbance was measured by a spectrophotometer (LabSystems IEMS Reader MF, Labsystems/Thermo Scientific, Helsinki, Finland) using 450 nm as the primary wavelength and 620 nm as reference wavelength.

### Dot blot assay

After an initial optimization using serum samples from healthy cats, two FMC cell lines with known HER2 expression status and three antibodies against HER2, detection and quantification of sHER2 levels were performed in the sera of 67 cats. Briefly, after drawing a 1 cm square grid on a nitrocellulose membrane (Protan - BA83, Whatman GmbH, Dassel, Germany) with a pencil, 1 μl of each serum sample was spotted at the center of each square, allowing the evaluation of 40 sera per membrane (8 cm x 6 cm). The membranes were then dried at 37°C for an hour, before being washed and blocked with 0.1M Tris-buffered saline buffer containing 0.1% Tween 20 and 1% bovine serum albumin (TBST-BSA, pH 8.0), for 30 min at room temperature (RT). Then, blots were incubated during 90 min at RT, with an anti-HER2 antibody (clone SP3, dilution 1:1000, Zytomed, Berlin, Germany) that specifically recognizes the extracellular domain of HER2 [46, 47]. Later, membranes were washed for 30 min with TBST (3 × 10 min) and incubated for 30 min at RT with an appropriate secondary antibody (1:100,000; goat anti-rabbit IgG horseradish peroxidase conjugated - HRP, Southern Biotech, Birmingham, USA). The presence of HER2 in serum was revealed by the use of an enhanced chemiluminescence detection kit (Clarity Western ECL Substrate, Bio-rad, California, USA), and a semi-quantitative analysis was performed by comparing the signal intensity obtained in serum dots to the signal intensities of crescent concentrations of rHER2-ECD (5, 10, 15, 25, 50, 75 and 100 ng/ml, eBioscience, San Diego, USA) diluted in 30% BSA-Tris-buffered saline plus 0.05% Tween 20 (TBST) and spotted in the first line of the nitrocellulose membrane. Serum HER2 levels were scored by two independent observers and, discordant interpretations were discussed to reach a consensus. All serum samples were evaluated in three independent experiments.

Finally, two antibodies raised against different regions of the intracellular domain of HER2 receptor were also used to determine whether this domain is present in the soluble HER2 forms, thus detectable in the serum of cats (clone A0485, dilution 1:3500, Dako Denmark A/S, Glostrup, Denmark and clone 4B5, dilution 1:20, Ventana Medical Systems Inc., Tucson, AZ, USA).

### Western blotting analysis

To evaluate antibody specificity, two feline serum samples with distinct HER2 levels previously determined by the Dot blot assay, were probed with the anti-HER2 monoclonal antibody raised against the ECD (clone SP3). The molecular weight of the sHER2 fragments were compared with the band of recombinant human HER2-ECD, and with the bands detected in whole cell extracts of human HER2-overexpressing breast cancer cell line (SKBR3, ATCC, Manassas, Virginia, USA) and a feline HER2-positive mammary carcinoma cell line (FMCp, kindly provided by Prof. Nobuo Sasaki, Tokyo University, Japan).

SKBR3 and FMCp cells grown in 30mm dishes were washed three times with phosphate-buffered saline (PBS) and lysed in radioimmunoprecipitation assay (RIPA) buffer (25 mM Tris, pH 8.2; 150 mM NaCl; 0,5% NP40; 0,5% sodium deoxycolate; 0,1% SDS) supplemented with the following protease and phosphatase inhibitors: cOmplete, Mini, EDTA-free (Roche), Phosphatase Inhibitor Cocktail Set V, 50x (Calbiochem, San Diego, USA) and PhoStop (Roche, Basel, Switzerland). After lysis, whole cell extracts were boiled at 95°C for 15 min, centrifuged at 14,000 g for 2 min at 4°C and stored at −80°C.

The diluted rHER2-ECD, the whole cell lysates and the serum samples were fractioned by 7.5% SDS-PAGE and electrophoretically transferred to a nitrocellulose membrane with 0.2 μm pore diameter (Whatman Schleicher & Schuell, Whatman GmbH, Dassel, Germany). The immunoblot was initially blocked using 2.5% (w/v) bovine serum albumin (BSA, Sigma) in TBST, to inhibit non-specific binding. For immunoblot analysis, membranes were then incubated with the primary and secondary antibodies used in the Dot blot assay. Signal intensity of reactive bands was detected by autoradiography using an enhanced chemiluminescence detection kit (Luminata Crescendo, ECL detection system, Millipore, Darmstadt, Germany).

### Statistical analysis

All statistical analyses were carried out using the Statistical Package for the Social Sciences for Windows software (SPSS, version 21.0, IBM, Armonk, New York, USA) and a two-tailed *p* value less than 0.05 was considered statistically significant.

After testing for normality, the Wilcoxon test was used to compare the sHER2 levels between cats with mammary carcinoma and healthy ones, and also to compare sHER2 levels and tissue HER2 status among cancer group cats. Receiver-operating characteristics (ROC) curves were performed to choose the optimal cutoff value for the ELISA and Dot blot assay, and to determine the sensitivity and specificity of both techniques using IHC as a gold standard technique. The concordance between the three assays was estimated by the Kappa test and correlations were evaluated by Spearman's rank correlation coefficient. The Fisher's exact test was used to assess the associations between sHER2 levels/tumor HER2 status and the clinicopathological features (breed, reproductive status, progestogens administration, prescribed treatment, number and location of tumor lesions, primary tumor size, lymph node status, stage of disease, histopathological classification, malignancy grade, presence of necrotic areas within the neoplasia, lymphatic vessel invasion by tumor cells, lymphocytic infiltration, cutaneous ulceration, Ki-67 index, HER2, ER and PR status). Whenever a cat showed multiple mammary tumors, the carcinoma with higher HER2 score (assessed by IHC), was the one selected for further studies. In cats with two or more mammary carcinomas showing equal HER2 scores, the lesion with higher malignancy grade and size was selected for statistical studies, since these two features have previously been associated with poor prognosis [48, 49]. Animals were also grouped by the tumor size, according to the TNM classification (< 2 cm; 2-3 cm; > 3 cm).

Overall survival (OS) period was defined as the time elapsed between the initial diagnosis and the death/euthanasia due to tumor metastasis. Disease-free survival (DFS) time was calculated from the date of surgery to the date of relapse (local, in other mammary gland or in distant organs) or death from cancer-related causes. Survival curves were estimated using the Kaplan-Meier method and the Log-rank test was used to compare the outcome (OS median and DFS median), regarding sHER2 levels and tumor HER2 status. For OS analysis, animals that died from a disease unrelated to mammary tumors or were lost during the follow-up were excluded.
